# Evaluation of the stability of tigecycline in elastomeric infusion devices used for outpatient parenteral antimicrobial therapy

**DOI:** 10.1093/jacamr/dlaf074

**Published:** 2025-05-13

**Authors:** Zenaw T Wolie, Sean Unwin, Andrew Burke, Hayoung Won, Steven C Wallis, R Andrew Seaton, Mark Gilchrist, Jason A Roberts, Fekade B Sime

**Affiliations:** Faculty of Medicine, Centre for Clinical Research, The University of Queensland, Building 71/918 RBWH Herston, Brisbane, Queensland 4029, Australia; Department of Pharmacy, College of medicine and health Sciences, Debre Markos University, Debre Markos, Ethiopia; Infection Management Services, Metro South Health, Princess Alexandra Hospital, Brisbane, Queensland, Australia; Faculty of Medicine, Centre for Clinical Research, The University of Queensland, Building 71/918 RBWH Herston, Brisbane, Queensland 4029, Australia; Thoracic Medicine, The Prince Charles Hospital, Brisbane, Queensland 4032, Australia; Faculty of Medicine, Centre for Clinical Research, The University of Queensland, Building 71/918 RBWH Herston, Brisbane, Queensland 4029, Australia; Faculty of Medicine, Centre for Clinical Research, The University of Queensland, Building 71/918 RBWH Herston, Brisbane, Queensland 4029, Australia; Department of Infectious Diseases, Queen Elizabeth University Hospital, Glasgow, UK; OPAT Initiative, British Society for Antimicrobial Chemotherapy (BSAC), Birmingham, UK; OPAT Initiative, British Society for Antimicrobial Chemotherapy (BSAC), Birmingham, UK; Department of Pharmacy/Infection, Imperial College Healthcare NHS Trust, London, UK; Department of Infectious Diseases, Imperial College London, London, UK; Faculty of Medicine, Centre for Clinical Research, The University of Queensland, Building 71/918 RBWH Herston, Brisbane, Queensland 4029, Australia; Herston Infectious Diseases Institute (HeIDI), Metro North Health, Brisbane, Queensland, Australia; Departments of Pharmacy and Intensive Care Medicine, Royal Brisbane and Women’s Hospital, Brisbane, Queensland 4029, Australia; Division of Anaesthesiology Critical Care Emergency and Pain Medicine, Nîmes University Hospital, University of Montpellier, Nîmes 30029, France; Faculty of Medicine, Centre for Clinical Research, The University of Queensland, Building 71/918 RBWH Herston, Brisbane, Queensland 4029, Australia

## Abstract

**Background:**

Tigecycline is increasingly being considered in outpatient parenteral antimicrobial therapy (OPAT) programmes given its spectrum of activity; however, stability data are lacking, necessitating further study.

**Objective:**

To assess tigecycline stability in elastomeric infusers under OPAT conditions, following the UK Yellow Cover Document (YCD) stability testing guidelines.

**Methods:**

Tigecycline was reconstituted with normal saline in Leventon Dosi-Fuser and Baxter-LV10 infusers at doses of 50, 100 and 200 mg in 240 mL. Additionally, a tigecycline intermittent infusion dose (50 mg/100 mL) was reconstituted in Baxter-SV100 infusers. The infusers were stored under refrigerated storage (2°C–8°C) for 7 days, followed by exposure at an in-use temperature of 32°C for 24 h, or at 25°C for 2 hours for the intermittent infusion. Stability was evaluated using a stability-indicating assay, pH measurement, subvisible particle count and visual inspection as per the YCD.

**Results:**

After 7 days of refrigeration followed by 24 h exposure to 32°C, the mean ± SD percentage of tigecycline remaining was 97.9 ± 0.6, 97.3 ± 0.6 and 95.4 ± 0.8 for the Baxter LV10 devices, and 97.2 ± 0.3, 96.9 ± 0.5 and 95.8 ± 0.8 for Dosi-Fuser devices at the low, intermediate, and high dose levels, respectively. For intermittent infusion in Baxter-SV100 devices, the mean ± SD percentage remaining after 7 days of refrigerated storage followed by 2 h at 25°C was 99.7 ± 0.2.

**Conclusions:**

Tigecycline meets the UK YCD criteria of ≤5% degradation limit, indicating its suitability for both intermittent and continuous 24-h infusion in OPAT programs.

## Introduction

Tigecycline, the first glycylcycline antibiotic within the tetracycline class, is a broad-spectrum antibiotic that works by inhibiting bacterial protein synthesis.^1,2^ Its spectrum activity covers a wide range of Gram-positive and Gram-negative organisms, including MRSA, ESBL-producing Enterobacterales, and certain carbapenem-resistant organisms.^3–6^ The US FDA and European Medicines Agency have approved tigecycline for the treatment of complicated skin and soft tissues infections (cSSTIs), complicated intra-abdominal infections (cIAIs), and community-acquired pneumonia.^3,7,8^ Additionally, it has been used off-label to treat various infections, including hospital-acquired pneumonia, ventilator-associated pneumonia, and febrile neutropenia.^8,9^

Tigecycline has been increasingly used in outpatient parenteral antibiotic therapy (OPAT) for treating complex infections caused by multi-resistant organisms, including bone and joint infections, intra-abdominal infections, non-tuberculous mycobacterial infections and parapharyngeal abscesses.^5,6^ The dosing regimen for tigecycline in OPAT is tailored to individual patient tolerability and their capacity for self-administration. Consequently, some patients may receive it twice daily, while others might be prescribed a once-daily regimen or continuous infusion (CI). Although tigecycline has long half-life that may allow once-daily intermittent dosing in OPAT settings, infusion related adverse effects such as nausea, vomiting, and diarrhoea are common with such high-concentration intermittent administrations.^5^ Slower infusion rates including CI administration are likely to minimize these adverse effects, such that there is growing interest in delivering tigecycline formulations by continuous 24-h infusion using elastomeric pumps. However, stability data for these formulations in OPAT settings have yet to be published. A previous study has shown that tigecycline remains stable for up to 48 h when reconstituted in normal saline or 5% dextrose and stored at refrigerated temperatures (2°C–8°C).^10^ However, data are scarce on its stability over extended periods at room or higher in-use temperatures exposures common in OPAT settings. Therefore, this study aims to evaluate the sequential stability of tigecycline in two elastomeric infusers, when stored refrigerated for 7 days followed by in-use temperature exposure at 32°C, including a semi-quantitative analysis of degradation products as specified in monographs and in accordance with the requirements outlined in the United Kingdom’s National Health Service (NHS) Yellow Cover Document (YCD) promoted by the British Society for Antimicrobial Chemotherapy drug stability testing programme.

## Materials and methods

### Materials

Tigecycline powder for injection (Tygacil^®^ 50 mg powder for reconstitution, AUST R 147450; Pfizer Australia Pty Ltd, Sydney, NSW; Batch number: ANCB/18, Exp: 03/2025) was used for the preparation of calibrators, quality control samples (QCs), and test solutions for the infusion devices. Three elastomeric infusion devices from different manufacturers were used: Baxter-LV10 (Baxter Healthcare Corporation, Deerfield, IL, USA; Lot #23F032, Exp:2026-06-01 with a nominal flow rate of 10 mL/h), Baxter-SV100 (Baxter Healthcare Corporation, Deerfield, IL, USA; Lot #22E028, Exp:2025-05-01 with a nominal flow rate of 100 mL/h) and Leventon Dosi-Fuser (L25915-250D1, Spirit Medical Ltd, Derbyshire, UK; Lot #211005L, with a nominal flow rate of 10.4 mL/h). The solvents used for the stability indicating method were HPLC-grade acetonitrile and methanol (LiChrosolv, Merck, Darmstadt, Germany). Potassium dihydrogen phosphate (Fisher Chemical, Fair Lawn, USA) and oxalic acid concentrate 0.1 M (Supelco, St Louis, USA) were used to prepare mobile phase A. Normal saline was sourced from Baxter Healthcare Pty Ltd, New South Wales, Australia.

### Preparation of tigecycline-filled infuser devices

Tigecycline powder for injection was reconstituted with normal saline by an experienced pharmacist adapting established procedures used in hospital pharmacies and in accordance with the product information for Tygacil^®^.^11^ Each vial was reconstituted with 5.3 mL of normal saline to prepare tigecycline solution at a concentration of 10 mg/mL. Then, 5 mL (equivalent to 50 mg) of this solution was transferred to a fluid bag containing the required volume of normal saline, in a Class II biosafety cabinet, to further dilute and create a solution of the nominal concentration. Three dosage levels of tigecycline were tested for 24-h CI administration: a low dose (50 mg in 240 mL), the standard adult maintenance dose (100 mg in 240 mL) and a high dose (200 mg in 240 mL). Additionally, a maintenance dose for intermittent infusion (50 mg in 100 mL) was tested. The solution prepared for each of these dosage levels was used to fill the devices to nominal volumes of 240 mL or 100 mL (for intermittent infusion), in triplicate for each device and concentration tested. The devices were then stored in a refrigerator at 2°C–8°C for 7 days. Following refrigeration, the devices were exposed to in-use temperature at 32°C or room temperature (RT) at 25°C (for the intermittent infusion only) for 24 h within a temperature-controlled incubator.

To allow sampling, the flow restrictors and in-line filters of the devices were removed, and the outflow line was clamped. Duplicate samples were collected from each device at 11 time points: 0, 24, 48, 96, 120 and 168 h under refrigeration, and 172, 176, 180, 188 and 192 h at 32°C. For intermittent infusion samples, collections occurred at similar time intervals during refrigeration, with additional samples taken at 169 and 170 h at 25°C. The entire study was conducted in a single batch, resulting in a total of 444 samples, with 0.5 mL aliquots being stored at −80°C for UHPLC-PDA assay. The pH of these samples was measured using a Horiba Scientific LAQUAtwin-pH-22 compact pH metre (Horiba, Kyoto, Japan). Visual assessments for colour, clarity and precipitation were conducted at each time point, adhering to Manual Visual Inspection guidelines from major pharmacopeias.^12^

Subvisible particles were evaluated using the Light Obscuration Particle Count Test with a Beckman Coulter HIAC 9703+ Liquid Particle Counter (Beckman Coulter Inc., CA, USA), in accordance with the standards set by the United States Pharmacopoeia (USP) and European Pharmacopoeia (EP) 2.9.19. HIAC analysis was performed at baseline, 24, 48 and 168 h during 7 days of refrigeration, and subsequently after 12 and 24 h at 32°C. Additionally, samples were analyzed after 1 and 2 h at 25°C for the intermittent infusion devices.

### Assay method and chromatographic settings

The stability-indicating assay method was developed and validated. Calibrators were prepared by diluting the tigecycline pharmaceutical product with normal saline to achieve the following concentration ranges: low calibrators at 20–250 mg/L, intermediate calibrators at 50–450 mg/L, intermittent calibrators at 100–600 mg/L, and high calibrators at 100–1000 mg/L. Similarly, QCs were prepared using the tigecycline powder for injection diluted with normal saline, resulting in the following nominal concentration concentrations: low at 208 mg/L, intermediate at 416 mg/L, intermittent at 500 mg/L, and high at 833 mg/L. All calibrators, QCs, and samples were stored in separate aliquots at −80°C until needed for analysis. On the day of analysis, calibrators, QCs, and samples were thawed at RT, vortex mixed and transferred (100 µL) to autosampler vials without further dilution or treatment.

The method was developed using a Nexera X2 ultra-HPLC—photodiode array (PDA) system, which included two LC-30AD pumps with degassers, a SIL-30AC autosampler, a CTO-30AD column oven, and an SPD-M30A photodiode-array detector, all controlled by LabSolutions software (Shimadzu, Kyoto, Japan). The stationary phase was a XTerra MS C18 (2.1 × 150 mm, 3.5 μm) analytical column (Waters, Milford, USA), preceded by a Security Guard ULTRA^™^ cartridge UHPLC C18 2.1 mm ID (Phenomenex, Torrance, USA) maintained at 40°C.

The mobile phase consisted of 78.5% of A (20 mM potassium phosphate aqueous buffer with 0.1% (v/v) oxalic acid concentrate, 0.1 M) and 21.5% of B (40% acetonitrile). This mixture was delivered isocratically at a flow rate of 0.32 mL/min over a 15-min run time. The PDA detector scanned wavelengths from 190 to 380 nm and tigecycline was quantified specifically at 245 nm. A 1 µL aliquot of the prepared sample was then injected onto the UHPLC-PDA system.

### Assay performance

Seven levels of calibrators were analysed for each of four calibration ranges: 20 to 250 mg/L, 50 to 450 mg/L, 100 to 600 mg/L and 100 to 1000 mg/L. The precision and accuracy for all calibrators involved were found to be within 1.7% and 1.3%, respectively. Specifically, the precisions of the slopes for the four linear calibration ranges were 0.4%, 0.7%, 0.1% and 0.9%, respectively. The means of the coefficients of determination (*r*²) were 0.9999, 0.9999, 0.9997 and 0.9999 across the four calibration ranges, respectively. To demonstrate intra-assay precision and accuracy, replicate analyses of QCs at three concentrations (low, nominal and high) were performed within a single batch. In contrast, inter-assay precision and accuracy were acquired over three separate batches. In both cases, precision and accuracy were maintained within a deviation of 1.8% across the four calibration ranges. The reproducibility and reliability of the method were further validated through incurred sample reanalysis. Notably, 100% of the reanalysis samples fell within a 2.5% deviation, with 74% of the samples exhibiting a deviation of 1.0% or less from the mean concentration.

### Tigecycline forced degradation test

A solution of tigecycline (800 mg/L) in normal saline was combined in a 1:1 ratio with saline and stress solutions, including 0.5 M HCl, 0.5 N NaOH and 3% H₂O₂, to conduct acid hydrolysis, base hydrolysis, and oxidative stress forced degradation tests, respectively. These solutions were stored at RT and in an oven at 50°C. The forced degradation process was quenched using the appropriate solutions after incubation periods of 0, 6, 24, 30 and 48 h at 50°C, as well as after 0 and 24 h at RT. Tigecycline and its degradation products were subsequently analysed using the developed and validated stability indicating method described elsewhere in this paper.

### Data analysis

Microsoft Excel 365 was used for data manipulation, analysis, and presentation, while selected plots were generated using GraphPad Prism version 10.1.2 (GraphPad Software LLC, MA, USA).

The cumulative amount of tigecycline that would be delivered to the patient during the infusion's running phase was estimated using the following equations:


(1) $$A=\overset{n}{\underset{i=1}{\sum }}\;\mathrm{Ci}\times Vi$$

(2) $$C_{i}=a\times t_{i}+b$$

(3) $$V_{i}=V_{i\text{-}1}-f\;\times (t_{i}--t_{i\text{-}1})$$

Where:

- A represents the estimated cumulative amount of tigecycline delivered to the patient.
- *C_i_* is the concentration of tigecycline predicted by a regression model at time *t_i_* (the time elapsed since the initiation of the running phase).
- *V_i_* is the volume of the solution remaining within the infuser at that time.
- The parameters a and b are the regression coefficients that relate the concentration of tigecycline to time.
- *V_i_*_−1_ is the volume at the previous time increment.
- *f* is the flow rate of the device.
- (*t_i_*−*t_i_*_−1_) represents the time interval between the current and previous measurements.

Calculations were performed in 1-min increments, with *i* ranging from 1 to *n* = 1440 min, corresponding to the 24-h infusion period.

## Results

### Colour, clarity and precipitation

No colour change or visible precipitation was observed in any samples or devices throughout the study under refrigerated and in-use conditions. All samples remained clear, with no turbidity observed throughout the study.

### Subvisible liquid particles

All samples subjected to subvisible particle analysis met the requirements of USP <788>, and EP 2.9.19 (i.e. the particle count for particles ≥ 10 μm should not exceed 25 particles per millilitre, and for particles ≥ 25 μm, the particle count should not exceed 3 particles per millilitre), with the exception of some samples collected at baseline (*t* = 0), where particle counts slightly exceeded the specified limits (Tables 1 and 2). However, the particle sizes for the intermittent infusion dose remained within the limits established by USP <788>, and EP 2.9.19 (Table 3).

**Table 1.** Subvisible particle cumulative counts per millilitre for particles ≥10 and ≥25 µm in Baxter LV10 infuser devices

Time (hours)	Baxter LV10 devices subvisible particles count (mean ± SD cc/mL)					
	Low dose (50 mg/240 mL)		Intermediate dose (100 mg/240 mL)		High dose (200 mg/240 mL)	
	≥10 µm	≥25 µm	≥10 µm	≥25 µm	≥10 µm	≥25 µm
0	38.11 ± 5.58	2.22 ± 0.96	33.34 ± 2.89	0.56 ± 0.96	37.56 ± 5.48	5.67 ± 3.76
24	8.34 ± 7.64	0.56 ± 0.96	11.11 ± 11.09	1.67 ± 2.89	6.89 ± 5.92	0.00 ± 0.00
48	6.67 ± 0.00	0.56 ± 0.96	6.11 ± 4.82	2.22 ± 2.55	7.22 ± 5.85	0.00 ± 0.00
168	11.11 ± 13.88	0.56 ± 0.96	14.44 ± 6.74	1.67 ± 1.67	9.44 ± 3.47	2.22 ± 0.96
180	0.56 ± 0.96	0.00 ± 0.00	2.22 ± 3.85	0.00 ± 0.00	2.78 ± 0.96	2.34 ± 1.55
192	1.11 ± 0.96	0.00 ± 0.00	8.33 ± 13.01	0.56 ± 0.96	3.89 ± 4.19	0.00 ± 0.00

cc/mL, cumulative counts/millilitre; SD, standard deviation.

**Table 2.** Subvisible particle cumulative counts per millilitre for particles ≥10 and ≥25 µm in Dosi-Fuser infuser devices

Time (hours)	Leventon Dosi-Fuser devices subvisible particles count (mean ± SD cc/mL)					
	Low dose (50 mg/240 mL)		Intermediate dose (100 mg/240 mL)		High dose (200 mg/240 mL)	
	≥10 µm	≥25 µm	≥10 µm	≥25 µm	≥10 µm	≥25 µm
0	27.78 ± 6.45	0.00 ± 0.00	34.89 ± 5.87	0.00 ± 0.00	65.33 ± 26.23	3.89 ± 6.74
24	10.55 ± 8.55	2.78 ± 2.55	3.33 ± 1.67	0.00 ± 0.00	8.33 ± 3.34	1.11 ± 1.92
48	6.67 ± 4.41	0.00 ± 0.00	4.45 ± 4.81	0.56 ± 0.96	2.78 ± 1.92	0.00 ± 0.00
168	3.89 ± 4.19	1.67 ± 1.67	7.22 ± 3.47	1.67 ± 2.89	8.89 ± 0.96	2.89 ± 1.07
180	1.67 ± 1.67	0.00 ± 0.00	1.11 ± 1.92	0.00 ± 0.00	5.00 ± 5.00	1.11 ± 1.92
192	3.33 ± 0.00	0.00 ± 0.00	1.67 ± 1.67	0.56 ± 0.96	0.56 ± 0.96	0.00 ± 0.00

cc/mL, cumulative counts/millilitre; SD, standard deviation.

**Table 3.** Subvisible particle cumulative counts per millilitre for particles ≥10 and ≥25 µm in Baxter SV100 devices

	Baxter SV100 subvisible particles count (mean ± SD cc/mL)	
	Intermittent infusion dose (50 mg/100 mL)	
Time (hours)	≥10 µm	≥25 µm
0	6.22 ± 4.35	2.89 ± 1.07
24	4.45 ± 2.55	0.56 ± 0.96
48	2.78 ± 1.92	0.56 ± 0.96
168	6.67 ± 2.89	2.78 ± 0.96
169	4.44 ± 3.47	2.78 ± 1.92
170	6.67 ± 2.89	2.22 ± 3.85

cc/mL, cumulative counts/millilitre; SD, standard deviation.

### pH changes

The changes in pH of the tigecycline solutions from baseline in LV10, Dosi-fusers, and SV100 elastomeric devices are summarized in Tables 4–6, respectively. Overall, the pH remained largely stable throughout the study, irrespective of the transition from refrigeration to in-use temperature conditions.

**Table 4.** Change in pH of tigecycline solution in Baxter LV10 elastomeric infusers filled with low, intermediate and high doses during refrigerated storage and in-use temperature exposure

Temperature condition	Time (hours)	Mean ± SD observed pH and change in mean pH from baseline by dose					
		Low dose (50 mg/240 mL)		Intermediate dose (100 mg/240 mL)		High dose (200 mg/240 mL)	
		Mean ± SD	Δ pH	Mean ± SD	Δ pH	Mean ± SD	Δ pH
Refrigerated storage (2°C—8°C)	0	5.32 ± 0.11	0.00	5.34 ± 0.02	0.00	5.26 ± 0.01	0.00
	24	5.42 ± 0.19	0.10	5.46 ± 0.07	0.12	5.37 ± 0.12	0.11
	48	5.53 ± 0.00	0.21	5.43 ± 0.13	0.09	5.32 ± 0.13	0.06
	96	5.52 ± 0.13	0.20	5.32 ± 0.11	−0.02	5.46 ± 0.03	0.20
	120	5.44 ± 0.08	0.12	5.23 ± 0.05	−0.11	5.37 ± 0.11	0.11
	168	5.30 ± 0.04	−0.02	5.30 ± 0.06	−0.04	5.32 ± 0.07	0.06
In-use temperature (32°C)	172	5.49 ± 0.08	0.17	5.33 ± 0.10	−0.01	5.29 ± 0.04	0.03
	176	5.40 ± 0.10	0.08	5.27 ± 0.07	−0.07	5.20 ± 0.03	−0.06
	180	5.51 ± 0.01	0.19	5.19 ± 0.05	−0.15	5.30 ± 0.07	0.04
	188	5.26 ± 0.05	−0.06	5.20 ± 0.07	−0.14	5.27 ± 0.09	0.01
	192	5.25 ± 0.04	−0.07	5.29 ± 0.09	−0.05	5.28 ± 0.07	0.02

pH, power of hydrogen; SD, standard deviation; ΔpH, change in pH from baseline calculated as: ΔpH = pH*_t_*−pH_0_, where pH*ₜ* is the pH at a given time (*t*) and pH₀ is the pH at the baseline (*t*₀).

**Table 5.** Change in pH of tigecycline solutions in Leventon Dosi-Fuser elastomeric devices filled with low, intermediate and high doses during refrigerated storage and in-use temperature exposure

Temperature condition	Time (hours)	Mean ± SD observed pH and change in mean pH from baseline by dose					
		Low dose (50 mg/240 mL)		Intermediate dose (100 mg/240 mL)		High dose (200 mg/240 mL)	
		Mean ± SD	Δ pH	Mean ± SD	Δ pH	Mean ± SD	Δ pH
Refrigerated storage (2°C—8°C)	0	5.22 ± 0.01	0.00	5.22 ± 0.1	0.00	5.26 ± 0.00	0.00
	24	5.51 ± 0.17	0.29	5.41 ± 0.16	0.19	5.48 ± 0.01	0.22
	48	5.48 ± 0.11	0.26	5.43 ± 0.11	0.21	5.38 ± 0.10	0.12
	96	5.60 ± 0.03	0.38	5.39 ± 0.16	0.17	5.35 ± 0.13	0.09
	120	5.62 ± 0.12	0.40	5.49 ± 0.16	0.27	5.31 ± 0.10	0.05
	168	5.46 ± 0.03	0.24	5.34 ± 0.08	0.12	5.42 ± 0.12	0.16
In-use temperature (32°C)	172	5.52 ± 0.05	0.30	5.35 ± 0.03	0.13	5.30 ± 0.04	0.04
	176	5.29 ± 0.05	0.07	5.35 ± 0.03	0.13	5.21 ± 0.08	−0.05
	180	5.51 ± 0.08	0.29	5.38 ± 0.09	0.16	5.37 ± 0.10	0.11
	188	5.40 ± 0.09	0.18	5.22 ± 0.05	0.00	5.24 ± 0.04	−0.02
	192	5.28 ± 0.14	0.06	5.32 ± 0.03	0.10	5.33 ± 0.11	0.07

pH, power of hydrogen; SD, standard deviation; ΔpH, change in pH from baseline calculated as: ΔpH = pH*_t_*−pH_0_, where pH*ₜ* is the pH at a given time (*t*) and pH₀ is the pH at the baseline (*t*₀).

**Table 6.** Change in pH of tigecycline solution in Baxter SV100 elastomeric devices filled for intermittent infusion doses during refrigerated storage and exposure to RT at 25°C

Temperature condition	Time (hours)	Mean ± SD observed pH and change in mean pH from baseline by dose	
		intermittent infusion dose (50 mg/100 mL)	
		Mean ± SD	Δ pH
Refrigerated storage (2°C–8°C)	0	5.28 ± 0.04	0.00
	24	5.36 ± 0.13	0.08
	48	5.32 ± 0.14	0.04
	96	5.48 ± 0.06	0.20
	120	5.52 ± 0.07	0.24
	168	5.45 ± 0.11	0.17
RT (25°C)	169	5.40 ± 0.11	0.12
	170	5.37 ± 0.11	0.09

pH, power of hydrogen; SD, standard deviation; ΔpH, change in pH from baseline calculated as: ΔpH = pH*_t_*−pH_0_, where pH*ₜ* is the pH at a given time (*t*) and pH₀ is the pH at the baseline (*t*₀).

### Tigecycline chemical stability

The mean (SD) percentages of tigecycline remaining at each time point for all dose levels tested in the LV10 and Dosi-Fusor devices during refrigerated storage (2–8°C) and in-use exposure at 32°C or 25°C (for the intermittent infusion dose in Baxter SV100) are summarized in Tables 7 and 8. Notably, >95% of tigecycline remained intact across both tested conditions and infuser devices used.

**Table 7.** Percentage of tigecycline remaining during refrigerated storage (2 to 8°C) for 7 days (0 to 168 h) followed by in-use temperature (32°C) for 24 h

Storage condition		Baxter LV10 devices Tigecycline % remaining (mean ± SD)			Leventon Dosi-Fuser devices Tigecycline % remaining (mean ± SD)		
	Time (hours)	Low dose (50 mg/240 mL)	Intermediate dose (100 mg/240 mL)	High dose (200 mg/240 mL)	Low dose (50 mg/240 mL)	Intermediate dose (100 mg/240 mL)	High dose (200 mg/240 mL)
Refrigerated storage (2 to 8°C)	0	100.0 ± 0.0	100.0 ± 0.0	100.0 ± 0.0	100.0 ± 0.0	100.0 ± 0.0	100.0 ± 0.0
	24	99.8 ± 0.3	100.0 ± 0.3	99.9 ± 0.4	99.8 ± 0.2	100.2 ± 0.2	99.9 ± 0.3
	48	99.9 ± 0.5	100.0 ± 0.3	99.7 ± 0.5	99.3 ± 0.2	99.8 ± 0.4	99.9 ± 0.1
	96	99.9 ± 0.8	99.7 ± 0.5	99.4 ± 0.5	99.1 ± 0.3	99.9 ± 0.3	99.8 ± 0.6
	120	99.7 ± 0.6	99.7 ± 0.3	99.2 ± 0.8	99.2 ± 0.1	99.7 ± 0.3	99.7 ± 0.4
	168	99.7 ± 0.1	99.5 ± 0.3	99.2 ± 0.7	99.3 ± 0.2	99.4 ± 0.4	99.5 ± 0.5
In-use temperature(32°C)	172	99.8 ± 0.4	99.4 ± 0.4	98.8 ± 1.0	99.0 ± 0.3	99.2 ± 0.6	98.9 ± 0.6
	176	99.5 ± 0.5	99.1 ± 0.4	98.3 ± 0.8	98.8 ± 0.2	98.9 ± 0.4	98.7 ± 0.3
	180	98.9 ± 0.3	98.4 ± 0.5	97.6 ± 0.9	98.8 ± 0.2	98.5 ± 0.7	98.0 ± 0.4
	188	98.2 ± 0.4	97.7 ± 1.0	96.0 ± 0.6	97.8 ± 0.1	97.4 ± 0.4	96.8 ± 0.6
	192	97.9 ± 0.6	97.3 ± 0.6	95.4 ± 0.8	97.2 ± 0.3	96.9 ± 0.5	95.8 ± 0.8

SD, standard deviation.

**Table 8.** Percentage of tigecycline remaining during refrigerated storage (2 to 8°C) for 7 days (0 to 168 h) followed by exposure to 25°C for 2 h

		Baxter SV100 device Tigecycline % remaining (mean ± SD)
Storage condition	Time (hours)	intermittent infusion dose (50 mg/100 mL)
Refrigeration (2 to 8°C)	0	100.0 ± 0.0
	24	100.1 ± 0.7
	48	99.9 ± 0.3
	96	100.0 ± 0.3
	120	100.0 ± 0.1
	168	99.7 ± 0.1
Running phase (25°C)	169	99.8 ± 0.1
	170	99.7 ± 0.2

SD, standard deviation.

### Tigecycline degradation products

**Figure 1.**
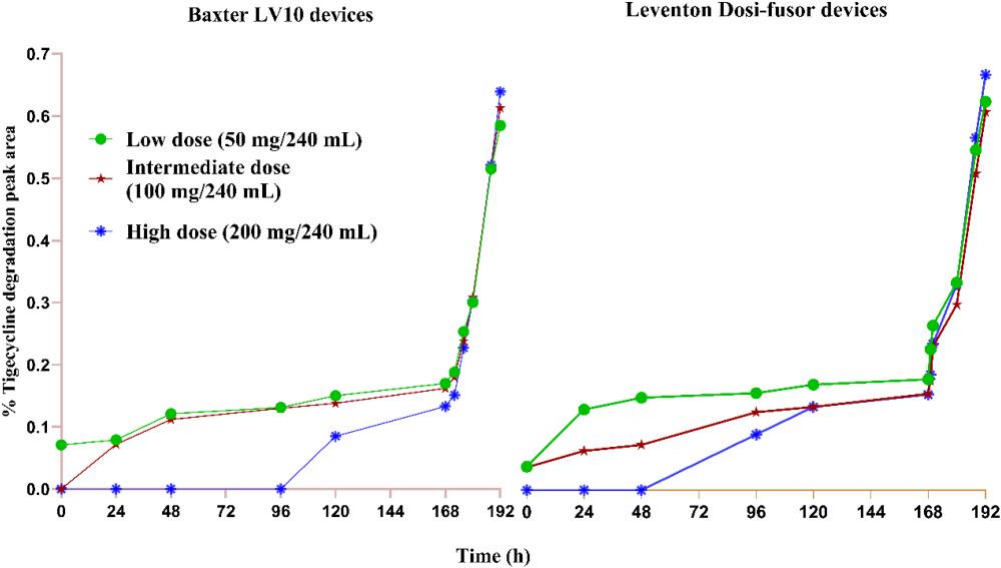
Percentage of cumulative peak area for degradation products of tigecycline relative to that of tigecyline during refrigerated storage, followed by 24 h of exposure to in-use temperature conditions.

The relative percentages of cumulative peak area of degradation products to that of tigecycline observed during the sequential stability study are depicted in Figure 1. Regardless of the dosage levels or infuser types employed, the overall mean degradation product peak area remained below 0.7% of the tigecycline peak area. During refrigeration, it remained steady at <0.2%, followed by a sharp increase during the running phase to just under 0.7%. Similarly, observed degradation peak area for the intermittent infusion was below 0.2% throughout the test conditions.

Results of tigecycline’s forced degradation tests under various stress conditions are summarized in Supplementary Figures S1 and S2 (available as Supplementary data at *JAC-AMR* Online). Tigecycline remains relatively stable at RT after reconstitution with saline/control, with approximately 20% degradation observed after 48 h. However, at elevated temperatures (50°C), tigecycline exhibited significant degradation, particularly under basic conditions, where nearly complete breakdown was observed. At RT, the amount of tigecycline remaining ranges from 79% (under basic stress conditions) to 96% (under saline/control, acidic and oxidative stress conditions). In contrast, at 50°C, the remaining tigecycline was 66%, 65%, 6% and 1% in acidic, oxidative, saline/control, and basic stress conditions, respectively.

### Estimated amount of tigecycline delivered to the patient

**Figure 2.**
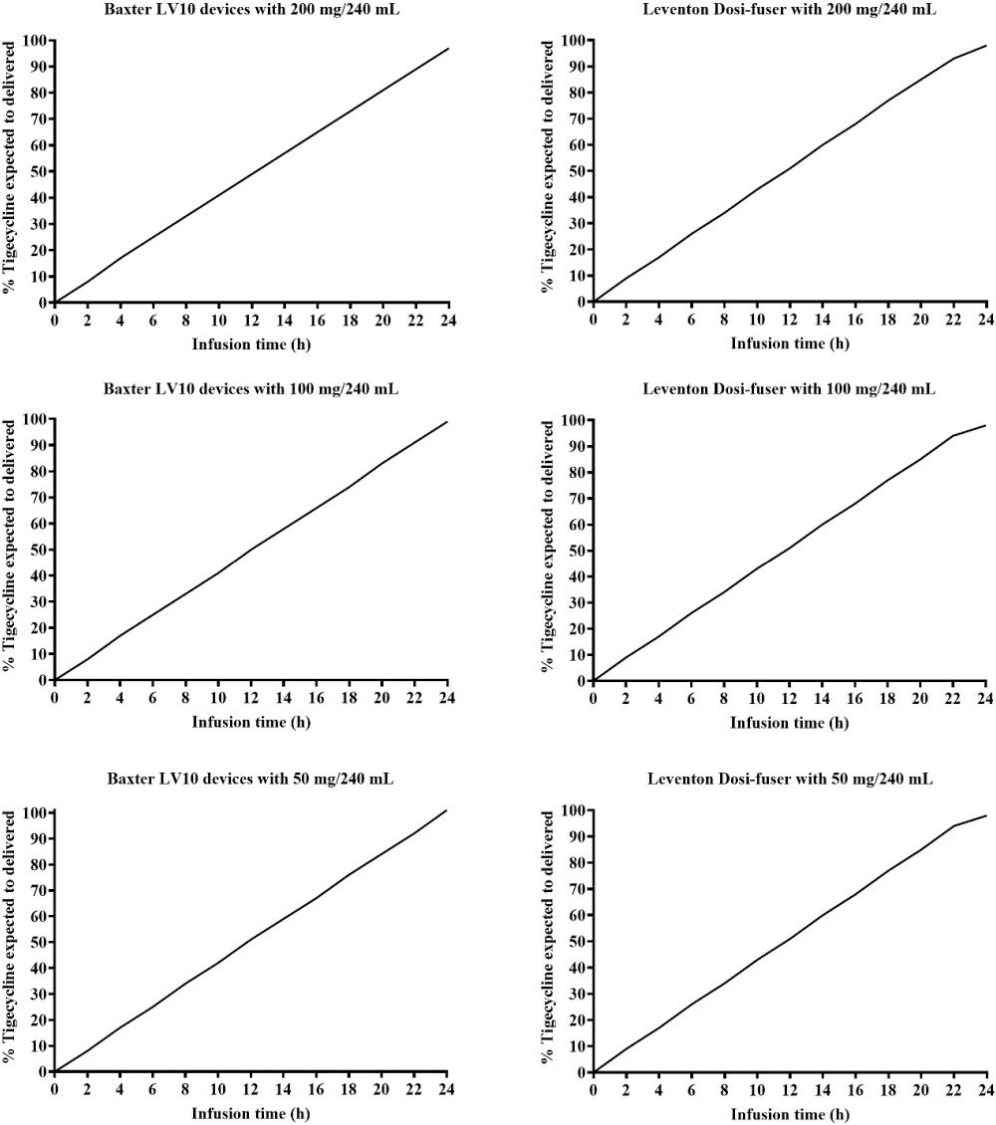
Estimated amount of tigecycline delivered to patients expressed as a cumulative percentage relative to the amount of drug in the elastomeric devices just after reconstitution at baseline (calculated at *t* = 0).

Figure 2 summarizes the estimated amount of tigecycline that would be delivered to the patient during the running phase, expressed as a percentage of the baseline amount (calculated at time 0), across various dose levels and devices. Assuming nominal flow rates are maintained, the percentage of the dose delivered for both devices ranged from 97% to 101%, as calculated based on equations 1–3.

## Discussion

This study investigated the stability of tigecycline when reconstituted with normal saline in elastomeric infusion devices at three different dose levels, following the stability testing framework outlined in the UK NHS YCD^13^ and promoted by the BSAC OPAT drug stability programme. The findings demonstrated that tigecycline is highly stable in clinically relevant storage and in-use conditions, making it suitable for both intermittent and CI in OPAT using elastomeric devices.

The physical stability of tigecycline was evidenced by consistent colour, clarity, absence of visible precipitation, and lack of turbidity—all essential indicators of drug safety, uniform appearance, and overall solution integrity. Although previous published data on tigecycline stability in saline solution is limited, stability in other aqueous solutions used for peritoneal dialysis (1.5% glucose, 7.5% icodextrin and 1.5% glucose pH neutral) is consistent with our findings.^14^

Additionally, tigecycline met USP <788 > and BP standards for subvisible particle counts under both refrigerated and in-use conditions. While particle counts from samples taken immediately after reconstitution were slightly higher, likely due to early samples before complete dissolution, the overall particle count remained within acceptable limits over time, affirming minimal risk of adverse events from particulate exposure. This stability is crucial for infusions intended to remain in a device for extended periods, as particulate accumulation could pose risks such as inflammation (phlebitis) and line blockage.^15^

The pH levels of tigecycline saline solutions remained stable, unaffected by dose variations or temperature changes between refrigeration and in-use conditions. Consistent pH is important, as fluctuations in pH conditions could affect drug stability and patient comfort upon administration.^16^ As indicated by the stable pH and measured concentration in this study, tigecycline demonstrated chemical stability in normal saline solutions. The percentages of tigecycline remaining after sequential exposure to refrigerated and in-use temperatures were consistently found to be greater than 95% across all tested dose levels and infusion devices. As illustrated in Figure 2, this stability is crucial for its effective use in OPAT, ensuring consistent and near-complete drug delivery during prolonged administration. Tigecycline’s chemical stability exceeding 95% in normal saline can be attributed to its chemical properties and the nature of the saline solution.^17^

Normal saline has a pH of approximately 5.5^18^ that is conducive for tigecycline stability, avoiding extreme pH conditions that could trigger major tigecycline degradation pathways such as oxidation (linked to the presence of a phenol moiety in its chemical structure) and epimerization, a chemical rearrangement that diminishes tigecycline's antimicrobial potency.^17^ In neutral or slightly acidic environments, the phenolic group in tigecycline remains protonated (-OH), making it less susceptible to oxidation that can lead to molecular degradation.^19^ While low pH can increase epimerization,^20^ the pH of normal saline is not low enough to significantly trigger this pathway. Additionally, tigecycline marketed as TYGACIL^®^ contains lactose monohydrate, which acts as a stabilizer in acidic environments, further reducing degradation pathways through epimerisation.^20^

In contrast, tigecycline is less stable in basic solutions where the pH exceeds the pKa of the phenol group.^19,21^ In these conditions, the phenolic group in tigecycline deprotonates, making it highly reactive towards oxygen and leading to faster degradation. Interestingly, at reduced pH levels (pH < pKa of the phenolic group), oxidation becomes less of a concern, and tigecycline’s oxidative degradation decreases as the pH is lowered.^19,20^ Therefore, congruent with our pH data (Tables 4 and 5), the risk of both oxidation and epimerisation is likely negligible when tigecycline is reconstituted with normal saline.

Clinically, tigecycline's stability in normal saline has significant implications for its utility in OPAT as it enables CI administration thereby minimising infusion related adverse drug reactions. There is high interest in the use of tigecycline in OPAT practice due to its broad-spectrum efficacy against MDR Gram-positive and Gram-negative pathogens^22^ that cause severe infections requiring prolonged parenteral antibiotic therapy. These include cSSTIs and cIAIs, and off-label use in challenging infections such as bone and joint infections.^5^

Clinical evidence supports tigecycline’s effectiveness in OPAT for complex infections. For instance, a study reported a 76% cure rate in patients with bone and joint infections and intra-abdominal infections, though some cases experienced adverse effects and treatment failures, underscoring the importance of careful patient selection and close monitoring.^5^ Tigecycline also provides a valuable treatment options when facing a true penicillin allergy scenario; for example, a case report has shown satisfactory patient recovery from an intra-abdominal abscess in a patient with a beta-lactam allergy after empiric broad-spectrum treatment with a standard dose of tigecycline.^23^ Furthermore, it offers a viable carbapenem-sparing option in polymicrobial infections involving drug-resistant Gram-negative organisms, such as Carbapenem-Resistant Enterobacterales, Acinetobacter baumannii, and Stenotrophomonas maltophilia strains.^24,25^ It also has an important OPAT role in MDR mycobacterial infections, albeit use is often limited by toxicity.

Of note, tigecycline has some clinical limitations that should be considered for its judicial use. First, the use of tigecycline in bloodstream infections is controversial due to its low serum concentrations and in most settings not recommended in routine clinical practice.^26^ Second, tigecycline has no reliable activity against Pseudomonas aeruginosa due to intrinsic resistance mechanisms such as efflux pumps and reduced outer membrane permeability, which may cause cross-resistance with other antibiotics.^27,28^ Third, gastrointestinal (GI) side effects such as nausea, vomiting, or diarrhoea, and infusion related side effects such as swelling, tenderness or pain at the injection site may occur. GI side effects may be minimized by taking food few hours prior to drug administration (e.g. 2 h prior to administration.^29^ Nevertheless, in practice this may not always work without concomitant use of antiemetics. On the other hand, slowing the infusion rate can help reduce infusion-related adverse events.^30^ Close patient monitoring and appropriate clinical adjustments are essential to maximize treatment success and ensure optimal outcomes.

In conclusion, the study supports the stability of tigecycline in normal saline for both intermittent and continuous 24-h infusion in OPAT settings using elastomeric infusion devices. For currently approved dosing regimens, further clinical studies and prospective clinical trials are warranted to explore whether continuous tigecycline administration reduces tigecycline side effects and optimizes therapeutic pharmacokinetic/pharmacodynamic (PK/PD) targets in OPAT settings.

## Supplementary Material

dlaf074_Supplementary_Data

## Transparency declarations

F.B.S. received research grant from Pfizer (2022–2023), Gilead Australia Fellowship (2023) and British Society for Antimicrobial Chemotherapy, BSAC (2021–2023). J.A.R. is a consultant/advisory board of Qpex (2022); Gilead (2022); Advanz Pharma (2022); Pfizer (2020); Sandoz (2020); Wolters Kluwer (2021); MSD (2021) and Summit Pharma (2021). J.A.R. received funding from Pfizer (2023); MSD (2022); Gilead (2022) and Cipla (2021) to speak at conferences. J.A.R. received industry grants from Pfizer (2023); QPEX (2021) and BSAC (2021–2023). M.G. is a consultant/advisory board of Pfizer (2023), Menarini (2023). M.G. is a Co-lead of the BSAC OPAT Initiative. M.G. is a consultant/advisory board of Pfizer (2023), Menarini (2023), Napp (2024). R.A.S. is a consultant/advisory board and/or speaker for Pfizer, Menarini, Shionogi and Napp (2023–2024). R.A.S. is a Co-lead of the BSAC OPAT initiative.

## Supplementary data

Supplementary Figures S1 and S2 are available as Supplementary data at *JAC-AMR* Online.
