# Supplementary material for: Evaluation of the stability of tigecycline in elastomeric infusion devices used for outpatient parenteral antimicrobial therapy
Source: JAC Antimicrob Resist. 2025 May 13;7(3):dlaf074. doi: 10.1093/jacamr/dlaf074 (PMC12070266; doi:10.1093/jacamr/dlaf074)
Supplement: dlaf074_Supplementary_Data [file dlaf074_supplementary_data.docx]

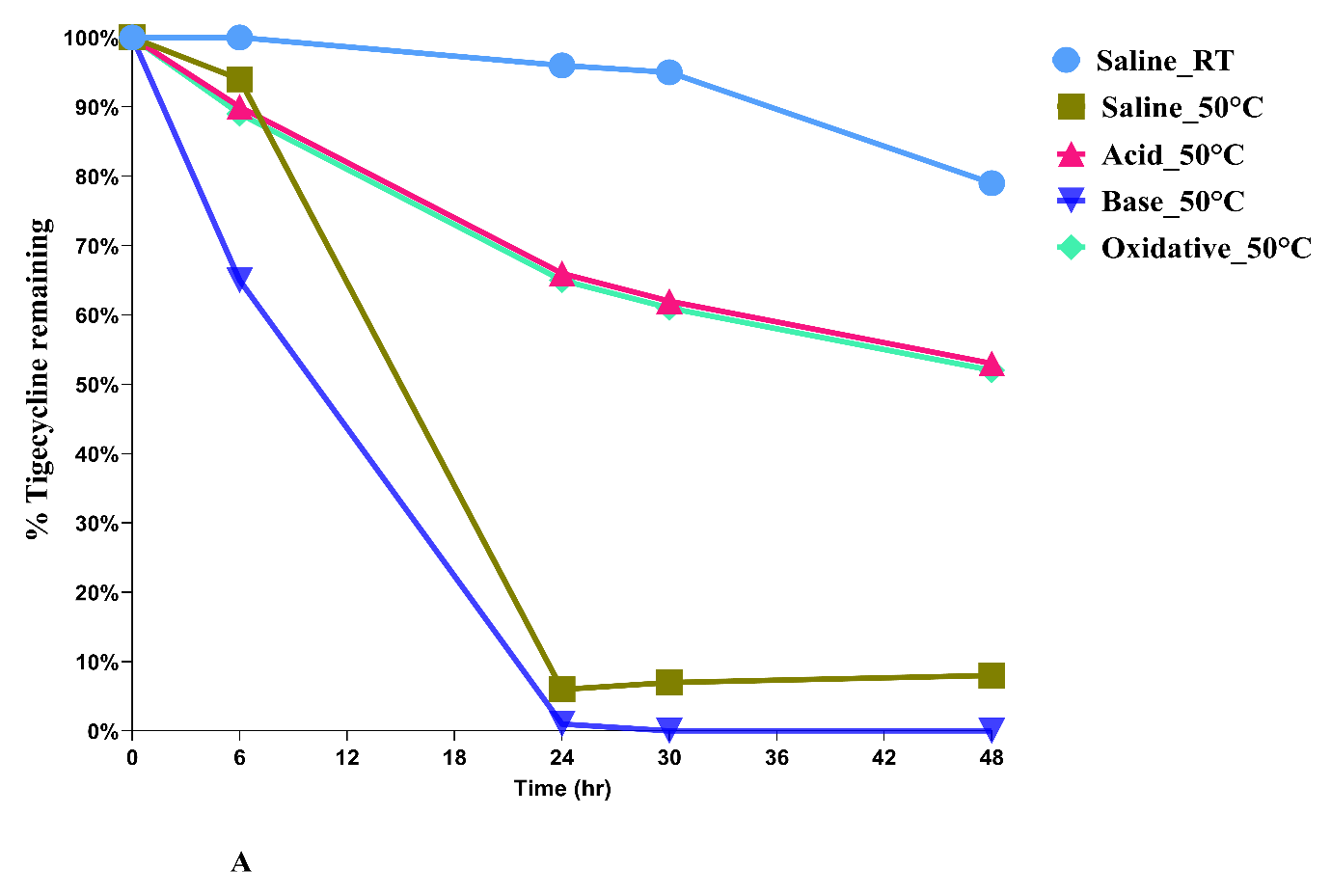


**Figure S1:** Time course of tigecycline percentage remaining under various stress conditions over 48 hours.


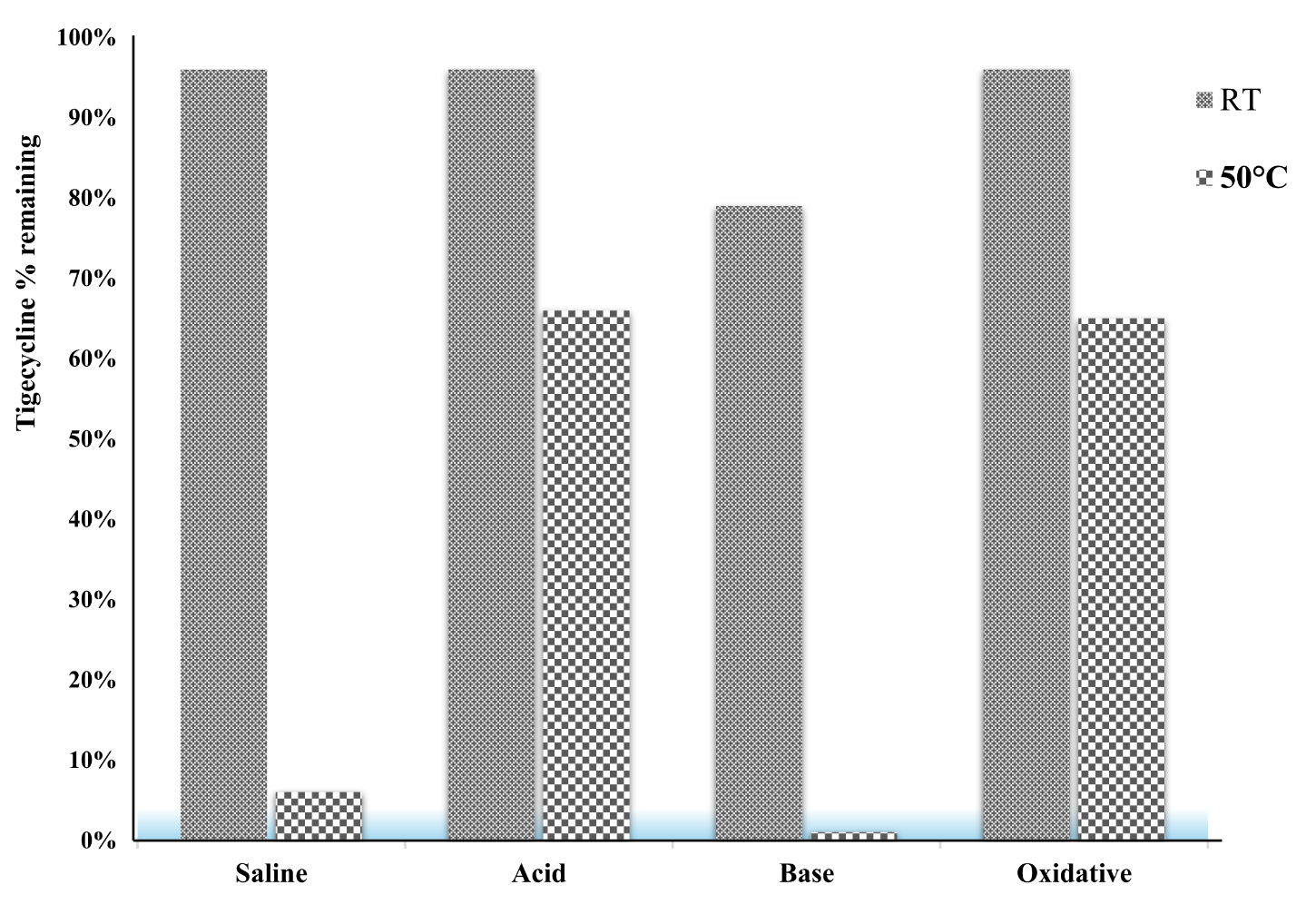


**Figure S2**: Comparison of tigecycline percentage remaining after 24 hours at room temperature (RT) and 50°C under various stress conditions.
